# Ionizing radiation-induced foci persistence screen to discover enhancers of accelerated senescence

**DOI:** 10.2147/IJHTS.S17076

**Published:** 2011-03-31

**Authors:** Edwardine Labay, Elena V Efimova, Benjamin K Quarshie, Daniel W Golden, Ralph R Weichselbaum, Stephen J Kron

**Affiliations:** Ludwig Center for Metastasis Research, University of Chicago, Chicago, IL, USA

**Keywords:** radiosensitizers, 53BP1, GFP, IRIF, tumor cell senescence, repurposing

## Abstract

Much like replicative senescence, the irreversible cell-cycle arrest induced by eroded telomeres, accelerated senescence occurs when replicative cells suffer irreparable DNA double-strand breaks (DSBs). Along with apoptosis and necrosis, senescence is a desirable outcome in cancer treatment with ionizing radiation (IR) or chemotherapy. In both normal and cancer cells, DSBs promote the assembly of IR-induced foci (IRIF), domains of modified chromatin that serve a key role in DNA damage signaling. IRIF persistence is a critical determinant of accelerated senescence, making drugs that promote persistent IRIF an attractive strategy to sensitize cancer to genotoxic therapy. As an IRIF reporter, we have expressed an inducible green fluorescent protein (GFP) fusion to the IRIF-binding domain (IBD) of 53BP1 (GFP-IBD) in the breast cancer cell line MCF7. Within minutes of exposure to IR, the GFP-IBD relocalizes to form fluorescent nuclear foci, which disperse within several hours. A pair of high-content screening assays for IRIF formation and persistence were established in multiwell plates based on imaging and quantifying GFP-IBD foci per Hoechst-stained MCF7 nucleus at 2 hours and 24 hours. Using the ataxia telangiectasia-mutated inhibitor CGK733 to block IRIF formation and the topoisomerase II inhibitor etoposide to prevent IRIF resolution, we obtained a Z′ >0.8 both for IRIF formation at 2 hours and IRIF persistence at 24 hours. Screening the diverse drugs and natural products in the National Cancer Institute Developmental Therapeutics Program Approved Oncology Drugs Set, the National Institutes of Health Clinical Collection, and the MicroSource Spectrum Collection yielded multiple hits that significantly delayed IRIF resolution. Secondary screening suggested some of these otherwise nontoxic drugs also enhance accelerated senescence, indicating strong potential for their repurposing as radiation sensitizers to improve the efficacy of cancer therapy.

## Introduction

Ionizing radiation (IR) induces DNA double-strand breaks (DSBs), which, if not repaired prior to cell division, result in cell death or irreversible cell-cycle arrest. This process is exploited medically in radiotherapy where radiation is focused on malignant tumors with the intent of eradicating cancer cells and sparing surrounding normal tissues. Although this strategy is often initially successful, tumors may recur locally or via distant metastasis. These cancers often display resistance by efficiently utilizing cellular pathways that mediate detection and repair of DSBs to recover from even the highest tolerated doses of IR. This has led to efforts toward discovery of agents that can frustrate radiation resistance and thereby enhance the cytotoxic effects of IR on tumors. Radiation sensitizers that permit lower total doses of IR and have greater effects would have a significant impact, particularly in the management of metastatic cancer.

The molecular analysis of DNA damage response has revealed a previously underappreciated role of chromatin modification and dynamics.^1–3^ Seconds after DNA damage, a cascade of protein modifications and recruitment ensues adjacent to each DSB. An early step in this process involves DNA damage recognition. This is mediated in part by phosphoinositide kinase-related kinases (PIKKs) such as ataxia telangiectasia-mutated (ATM), ATM- and RAD3-related (ATR), and DNA-activated protein kinase (DNA-PK). Multiple screens have identified inhibitors of PIKKs with potential as radiosensitizers.^4–8^ Once activated, ATM and the related PIKKs phosphorylate H2AX at DSBs, forming γH2AX (phosphorylated variant histone H2AX) foci, which serve as sites for further chromatin modifications and assembly of proteins with known roles in DNA damage repair and checkpoint signaling to form IR-induced foci (IRIF).^3,9,10^ Detailed genetic and molecular studies have characterized the assembly of IRIF and established a pathway from H2AX phosphorylation to recruitment and retention of ATM itself, MDC1, BRCA1, 53BP1, and other proteins.^1,3,9,10^ The modified chromatin domain can extend hundreds of kilobases away from break sites, increasing in size over time. Persistent DSBs may determine the antitumor effects of IR by inducing apoptosis, necrosis, mitotic catastrophe, or permanent growth arrest. The growth of persistent foci at unrepaired DSBs is likely essential for maintaining a G1 checkpoint signal.^11^ In turn, multiple studies have established the kinetics of appearance and resolution of γH2AX and IRIF as a proxy for DSB repair and radiation resistance.^12^ Importantly, the cytoxicity of IR can be augmented by blocking the assembly or function of IRIF.^8,13–15^

IRIF also form at eroded telomeres, and persistent IRIF serve a key role in directing both replicative and accelerated senescence.^16–18^ Although normal tissue senescence may underlie some of the side effects of genotoxic cancer therapy, accelerated senescence of cancer cells (therapy-induced senescence) has been proposed as a desirable outcome for cancer treatment. Senescent tumor cells may serve a positive role in slowing recurrence and promoting antitumor immune response.^19–26^ In order to determine whether or not accelerated senescence is a desirable clinical outcome of cancer therapy, it will be necessary to develop efficient and specific approaches to modulating relevant pathways in order to activate the response in a predictable fashion, highlighting candidate biomarkers and potential targets for intervention. Strategies to induce senescence in cancer cells for disease stabilization might provide future therapies complementary to existing interventions aimed at cell death. Senescence-targeted drug discovery could be accelerated by applying novel cell-based screening approaches to identifying and validating small-molecule promoters of cellular senescence as a route for disease stabilization. Cell-based screens can be used to interrogate senescence pathways to provide insights into the mechanisms of action of senescence promoters.

These considerations provide a strong rationale for targeting the accumulation or persistence of γH2AX or other IRIF markers as the basis of screens for genes and agents that alter the response to genomic damage, thus affecting cell death or cancer cell senescence.^27–29^ Indeed, we recently described using an IRIF reporter based on a green fluorescent protein (GFP) fusion to the IRIF binding domain (IBD) of 53BP1 (GFP-IBD)^30,31^ to examine determinants of IRIF formation and resolution. We found that combining the poly(ADP-ribose) polymerase inhibitor veliparib (ABT-888) with IR markedly enhances IRIF persistence. Within 4–7 days after IR, MCF7 cells treated with veliparib begin to display cellular markers of accelerated senescence, such as the characteristic large, flattened cell shape, increased p21^Cip1^ expression, and increased staining for senescence-associated β-galactosidase (SA-βGal).

Here, we report adapting the GFP-IBD IRIF reporter to enable a quantitative high-throughput screening (HTS) assay and using this tool to screen compound libraries for altered kinetics of IRIF formation and persistence in living cells. Control agents that prevent IRIF assembly or block IRIF resolution provided a reliable signal under screening conditions. In collections of approved and investigational drugs, we found a surprising number of compounds that scored as hits, suggesting high potential to identify practical, nontoxic radiosensitizers that might be rapidly translated to the clinic.

## Materials and methods

### Cell lines, constructs, and irradiation

The generation and characterization of the MCF7^Tet-On^ GFP-IBD cell line has been described.^31^ The cell line was maintained in high-glucose Dulbecco’s Modified Eagle Medium (Invitrogen Corporation, Carlsbad, CA) with 10% Tet system approved fetal bovine serum (FBS, Clontech, Mountain View, CA), 100 units/mL penicillin, and 100 μg/mL streptomycin at 5% carbon dioxide and 37°C. In all experiments, IR was delivered by a ^60^Co source (GammaCell, MDS Nordion, Ottawa, ON). The clonogenic assay, SA-βGal assay, and immunodetection of endogenous 53BP1 were performed as described previously.^31^

### High-content screening

MCF7^Tet-On^ GFP-IBD cells were plated in 2 × 10^4^ cells/well in 96-well black/clear thin-bottom tissue culture-treated imaging plates (Greiner #655090, Greiner BioOne, Monroe, NC) with 1 μg/mL doxycycline in the media. They were incubated for 48 hours for induction of GFP-IBD before being exposed to dimethyl sulfoxide (DMSO) with or without added compounds in a humidified incubator in 5% carbon dioxide at 37°C for 1 hour and then irradiated with 2 Gy or 6 Gy and returned to the incubator. On each plate, there was a column of eight vehicle-only control wells containing 0.5% DMSO, the final concentration of DMSO in all sample wells throughout the assay. Compounds were added directly at 1 μL/well from stock plates for 50 μM or diluted 1:8 in DMSO and then added at 1 μL/well for 6.25 μM concentrations. The topoisomerase II inhibitor etoposide was chosen for the positive control for foci persistence at 24 hours. The etoposide causing IRIF formation independently of IR treatment allowed us to use a single positive control with or without IR. At 2 hours or 24 hours post-IR, nuclei were stained using 5 μg/mL Hoechst 33342 (Sigma-Aldrich, St Louis, MO) in complete media, returned to the incubator for 15 minutes, and then imaged live or after fixation with 2% paraformaldehyde in PBS for 15 minutes, followed by two washes with PBS.

### Image acquisition and analysis

Image acquisition was performed using an ImageXpress Micro Cellular Imaging System (Molecular Devices, Sunnyvale, CA) equipped with a Nikon CFI S Plan Fluor ELWD 40 × /0.60 NA objective with correction collar and a Photometrics CoolSnap HQ camera in a 96-well format with four fields imaged per well. Semrock (Rochester, NY) BrightLine fluorescence filter sets were used to detect GFP-labeled IRIF (472/30 nm excitation, 520/35 emission) and Hoechst 33342-stained nuclei (377/50 excitation and 447/60 emission). Laser-based autofocus was performed in each well. IRIF were detected and quantified using the MetaXpress (v1.7) granularity application module, with automatic segmentation to define individual nuclei based on Hoechst fluorescence followed by counting distinct GFP fluorescent foci (measured as granules per cell) within each nucleus. In a typical experiment, 60–80 nuclei were present in an IR only-treated field, and four fields were measured per well, giving us 240–320 nuclei per compound for analysis. A threshold intensity of 50 gray levels above local background and a minimum width threshold of three pixels (1.1 μm) were used to distinguish presumptive IRIF from background noise. Insofar as newly formed and/or small foci are as small as one pixel (~0.4 μm) in apparent size, this threshold gated out bona fide IRIF that failed to grow by 2 hours or 24 hours, occasionally resulting in false identification as “foci suppressors”. Visual inspection of images for low- and high-scoring wells was thus used as a secondary screen to identify these and other artifacts. The maximum width threshold for foci of 19 pixels (6 μm) was chosen to include the largest foci observed. Data were normalized to foci number/cell in the 0.5% DMSO control matched for time point and irradiation dose, with fold change reported for each compound. A cutoff of 0.5-fold change in the average number of foci/nuclei was used as the criterion for foci suppression (which included both compounds that suppressed foci formation completely and compounds that inhibited normal development of foci) and 1.5-fold change for increased foci number and/or enhanced foci persistence.

Cytotoxicity was measured indirectly by counting nuclei present per field and using a cutoff of 0.5 for fold change in nuclei number compared with control matched for IR dose and time point for exclusion due to toxicity.

### Z′ factor analysis

For Z′ factor analysis, we preincubated 48 wells of two 96-well plates for 1 hour with DMSO alone, and the remaining 48 wells were treated for 1 hour either with 50 μM CGK733 (ATM inhibitor, Tocris Bioscience, Ellisville, MO) in DMSO or with 25 μM etoposide (topoisomerase II inhibitor, Sigma-Aldrich) in DMSO. Both plates were then irradiated with 6 Gy and incubated for 2 hours or 24 hours before analysis on the ImageXpress system as described previously. The Z′ factor was determined at 2 hours for CGK733 using a cutoff of 0.5-fold change from control and at 24 hours for etoposide using a cutoff of 1.5 by applying the equation Z′ = 1 − (3σ_p_ + 3σ_n_)/|μ_p_ − μ_n_|, where σ_p_ = standard deviation of the true positives, σ_n_ = standard deviation of the true negatives, μ_p_ = mean of the true positives, and μ_n_ = mean of the true negatives.^32^

### Compound libraries

The 89-compound National Cancer Institute Developmental Therapeutics Program (DTP) Approved Oncology Drugs Set, the 480-compound National Institutes of Health (NIH) Clinical Collection (BioFocus, South San Francisco, CA), and 1990 compounds of the Spectrum Collection (MicroSource, Gaylordsville, CT) were provided at a concentration of 10 mM DMSO in 96-well plates. To confirm selected hits, the compounds were obtained from independent commercial sources and then assayed at a range of concentrations for foci persistence and accelerated senescence, using methods previously described.^31^

## Results

### A high-content screening assay for IRIF persistence

**Figure 1:**
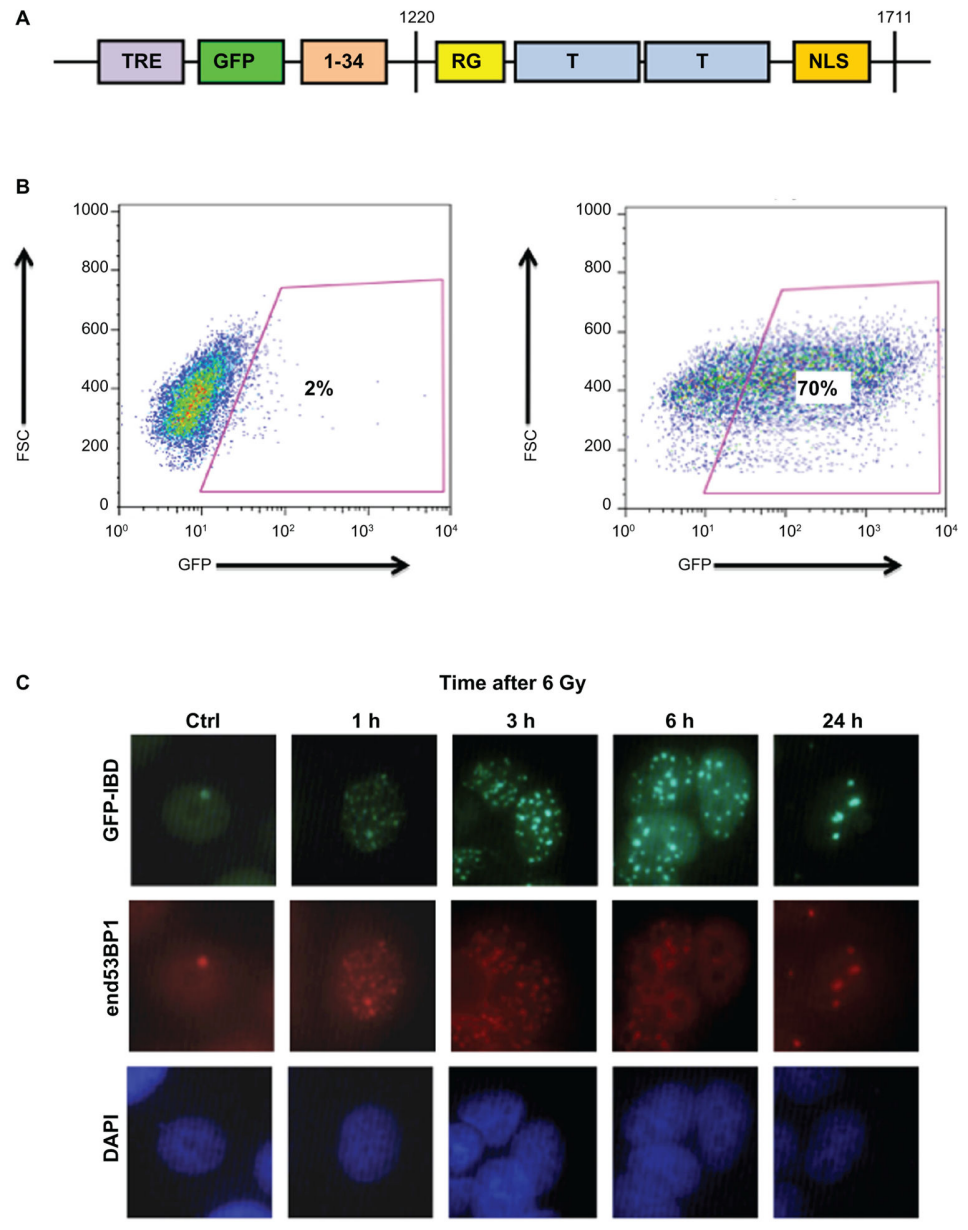
The green fluorescent protein ionizing radiation-induced foci-binding domain (GFP-IBD) construct expressed in cells functions as a reporter for DNA damage. **A**) The GFP-IBD reporter construct contains GFP fused to residues 1–34 and 1220–1711 of human 53BP1. The Tudor domains are placed 3′ of GFP and a tetracycline inducible promoter. **B**) The two panels show flow cytometry of MCF7^Tet-On^ GFP-IBD uninduced (control, 2% GFP-positive) and 48 hours after induction with 1 μg/mL doxycycline when 70% of cells scored as GFP-positive. **C**) After 48 hours of induction with 1 μg/mL doxycycline, MCF7^Tet-On^ GFP-IBD cells were irradiated with a single dose of 6 Gy. Immunofluorescence staining of endogenous 53BP1 shows colocalization with GFP-IBD at all time points.

To track DSB formation and resolution after irradiation, we used our previously described IRIF reporter GFP-IBD transduced into the MCF7 Tet-On advanced cell line (MCF7^Tet-On^, Clontech).^31^ The parent MCF7 cell line is deficient in caspase-3, leading to defects in apoptotic response, so the most confounding effects of apoptosis can be avoided. GFP is fused to the 53BP1 glycine rich motif (RG), tandem tudor domains (T), and nuclear localization signal (NLS)^30^ and expressed under inducible control of the tetracycline-responsive element (TRE) of the pLVX-Tight-Puro vector (Figure 1A). Nearly 70% of the MCF7^Tet-On^ cells expressed the GFP-IBD protein after 48 hours of induction with 1 μM doxycycline (Figure 1B), and most remained fluorescent past 72 hours. This fusion construct serves as a reliable reporter of 53BP1 relocalization to sites of DNA damage, colocalizing with endogenous 53BP1 following irradiation and disappearing as the IRIF resolve (Figure 1C). A similar construct has been used as a dominant negative to block 53BP1 binding and disrupt IRIF function,^33^ but endogenous 53BP1 foci formation and persistence were minimally affected by GFP-IBD under the conditions of our assay, indicating that the endogenous 53BP1 remains active.^31^

**Figure 2:**
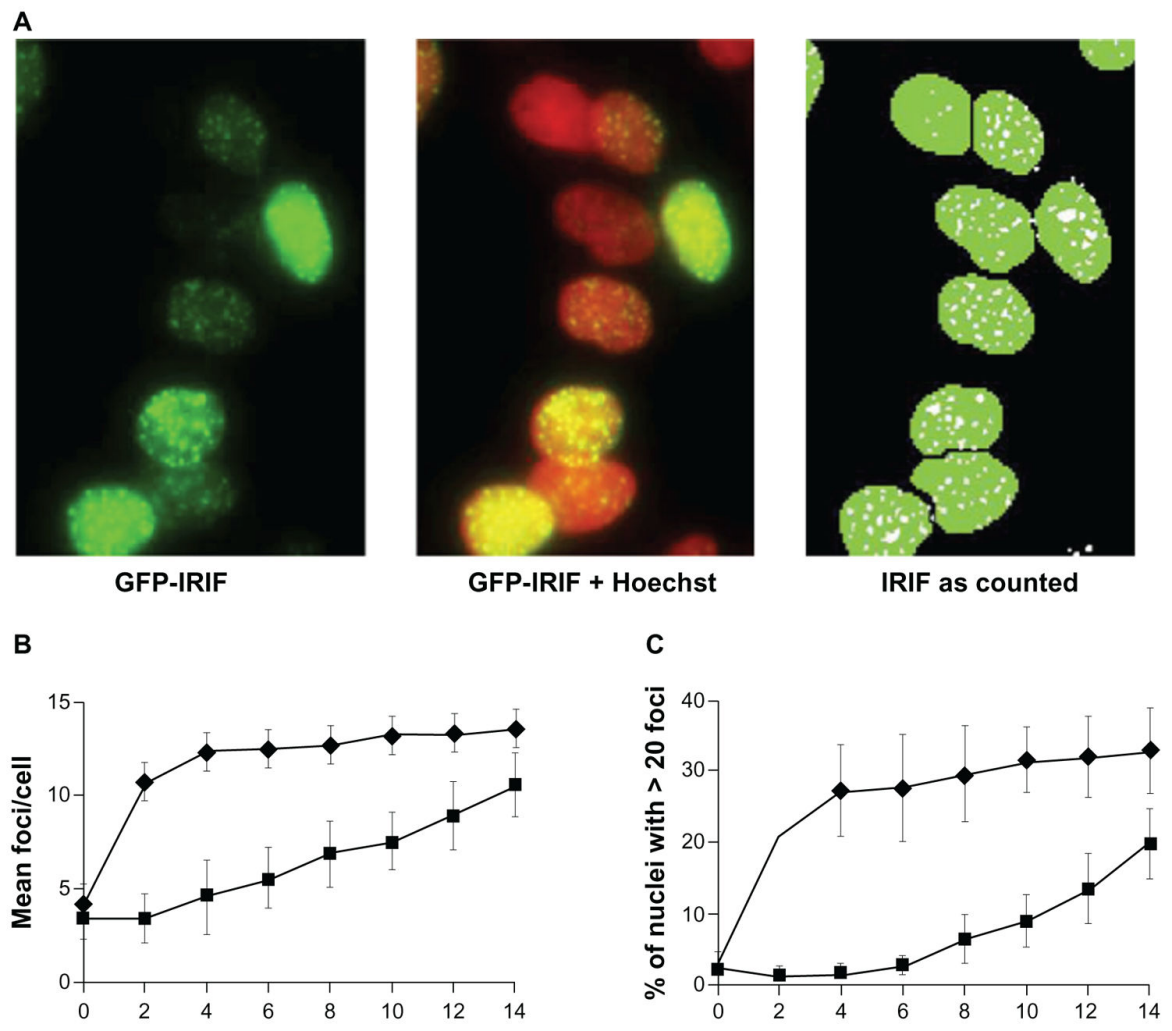
Optimizing quantification of development and resolution of ionizing radiation-induced foci (IRIF) in MCF7^Tet-On^ green fluorescent protein (GFP)-IBD. A threshold of 1.1 μm was set for the minimum width for foci to exclude background. After a 48-hour induction with doxycycline, cells were pretreated for 1 hour with 6.25 μM etoposide and then dosed with 6 Gy IR. Twenty-four hours later, cells were scanned and imaged with the ImageXpress system. **A**) Automated imaging of GFP-labeled IRIF (pseudocolored green) in the leftmost panel, with Hoechst 33342-stained nuclei (pseudocolored red) superimposed in the middle panel. The right panel shows the result of automated segmentation of nuclei (Hoechst) and foci (GFP) prior to quantification by the granularity module of the MetaXpress software. **B**) Average number of IRIF per cell was calculated from four fields/well in triplicate wells at 2 hours (upper line, diamonds) and 24 hours (lower line, squares) at IR doses from 2 Gy to 14 Gy. **C**) The percentage of cells with nuclei with >20 foci counted at 2 hours (upper line, diamonds) and 24 hours (lower line, squares) at IR doses from 2 Gy to 14 Gy. Based on these data, 6 Gy was selected as a screening dose.

GFP-IBD foci are detectable immediately after exposure to IR and then appear to grow in number and size for several minutes. Contributing factors may be continuing recruitment of GFP-IBD to modified chromatin immediately adjacent to breaks, as well as spreading of the chromatin modifications distally from the break site. Within the first hour, foci begin to disappear, likely reflecting rapid DSB repair by nonhomologous end joining. After a rapid phase, a slower rate of foci resolution ensues, continuing for at least 24 hours, during which time any remaining foci continue to become both brighter and larger. In order to evaluate alterations in the kinetics of foci assembly and both the fast and slow phases of resolution, assays were run at both the 2-hour and 24-hour time points. Foci numbers were determined using the granularity module in MetaXpress by adjusting nuclei segmentation and foci size thresholds empirically to obtain the most reproducible results using etoposide as the positive control, as outlined in Materials and methods (Figure 2A). Using this automated counting approach, we examined GFP-IBD foci numbers at 2 hours and 24 hours as a function of IR dose from 2 Gy to 14 Gy to identify optimal screening conditions. Prior analysis using confocal microscopy for foci counting^31^ demonstrated that with increasing dose, foci numbers increased while foci size decreased, eventually frustrating confident determination of foci numbers. Although the maximum number of foci that can be counted using confocal microscopy appears higher than when using the ImageExpress high-content screening tool, the trend is the same. We found that a dose of 6 Gy and imaging 2 hours post-IR may represent an upper limit for accurate foci counting, yielding 13 ± 2 foci. Above 6 Gy, plateaus in the average foci number and in the fraction of cells with >20 foci per nucleus were observed with increasing dose (Figure 2B and 2C). In turn, foci numbers and the number of cells with >20 foci per nucleus each decreased over 24 hours by 50% or greater up to a dose of 10–12 Gy. By 24 hours after a 6 Gy dose, the residual GFP-IBD foci decreased to 5 ± 2 per cell (Figure 2B), and the number of cells with >20 GFP-IBD foci was <5% of the total (Figure 2C). From our previous data,^31^ a dose of 6 Gy is also the point in the clonogenic assay of MCF7 at which cell survival changes slope as a regimen of higher lethality is entered. Though significantly higher than a standard radiation therapy fraction of ~2 Gy, this dose provided sufficient dynamic range to reliably detect suppression of IRIF formation at both 2 hours and 24 hours and increased IRIF persistence at 24 hours.

### Validation of high-content screening conditions

A challenging factor for high-content screening in detecting small molecules that alter IRIF function is that IRIF change in both size and number over time.^10,38^ In turn, agents that alter IRIF function not only affect kinetics of foci formation and resolution but also may affect the growth of foci (eg, PARP and HDAC inhibitors).^31^ Thus, an assay to detect modulation of IRIF must be sensitive to both changes in foci number and size of foci formed.

**Figure 3:**
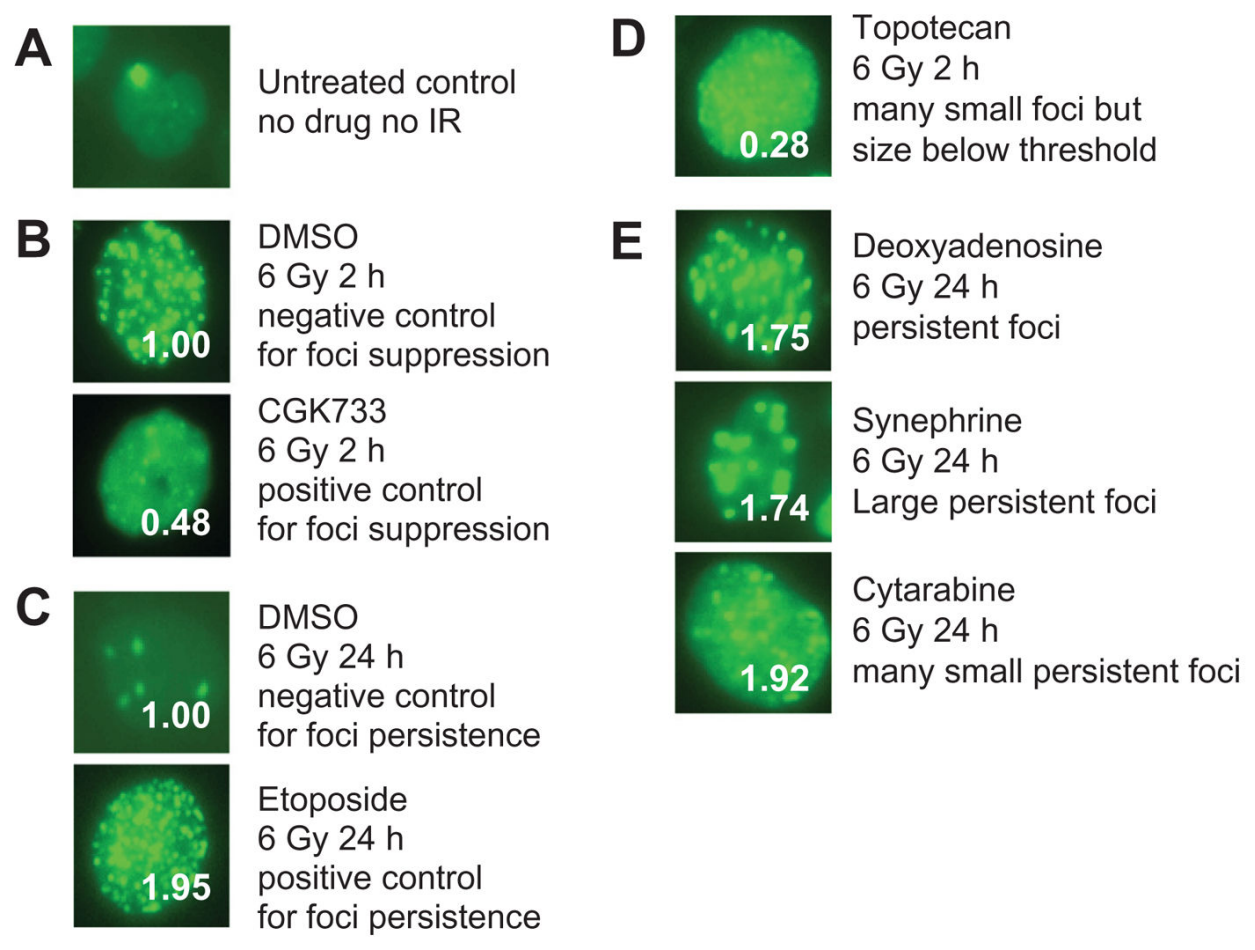
Patterns of foci modification. **A**) Most untreated cells typically have zero to two foci, with occasional cells with a higher number of foci. **B**) (Upper) The negative control for foci formation at 2 hours was exposure to 1-hour pretreatment with 0.5% dimethyl sulfoxide (DMSO) (vehicle), irradiation with 6 Gy, and imaging 2 hours post-ionizing radiation (IR). The resulting mean foci count was given a value of 1.00 for reference at the 2-hour time point. (Lower) A positive control for agents mediating suppression of foci formation is the ataxia telangiectasia-mutated (ATM), ATM- and RAD3-related (ATR) inhibitor CGK-733. At 2 hours after 6 Gy, CGK-733 yielded a value of 0.48, or 48% of 2 hours of negative control. **C**) (Upper) A negative control for foci persistence at 24 hours was exposure to 1 hour of pretreatment with 0.5% DMSO (vehicle), irradiation with 6 Gy, and imaging 24 hours post-IR. The foci count was assigned a value of 1.00. (Lower) As a positive control for agents promoting enhanced foci persistence, the topoisomerase inhibitor etoposide was used. etoposide yielded a score of 1.95 at 24 hours post-6 gy. **D**) Pretreatment with topotecan 1 hour before the 6 gy dose and imaging at 2 hours yields many very small foci that fall below the software threshold, giving an artifactual value of 0.28 for foci number. **E**) Hits that scored >1.5 in the screen for enhanced foci persistence displayed a range of foci phenotypes. Deoxyadenosine (1.75) foci appeared similar to control foci but in greater numbers, synephrine (1.74) foci were comparatively less abundant but proportionately larger and brighter, and cytarabine (1.92) yielded many small, persistent foci.

Examples of relevant phenotypes are shown in Figure 3. In the unirradiated control, most MCF7^Tet-On^ GFP-IBD cells display relatively homogeneous nuclear fluorescence and/or 0–2 GFP-IBD foci (Figure 3A). To evaluate effects on IRIF kinetics, we determined the mean count of GFP foci/Hoechst-stained nucleus by automated imaging, as outlined in Materials and methods, and normalized this value relative to the IR only control value at the same time point, which is assigned a value of 1.00. For each condition and treatment, the number after the condition or drug represents this normalized score (eg, Figure 3B and 3C). When irradiated cells treated with DMSO were examined 2 hours after 6 Gy irradiation, >20 GFP-IBD foci remained visible, and the ATM kinase inhibitor CGK733^8^ markedly suppressed foci formation to 0.48 (Figure 3B). At 24 hours after 6 Gy, <5 GFP-IBD foci remained in the irradiated cells treated with DMSO, but addition of the topisomerase II inhibitor etoposide significantly enhanced foci persistence to 1.95 (Figure 3C). Based on these data, we concluded that determination of foci per nucleus would be a satisfactory metric for screening. These conditions were then repeated in multiple wells of 96-well plates and analyzed with the ImageXpress Micro Cellular Imaging System using conditions optimized to allow reliable counting of foci numbers over a wide range of foci sizes. By testing a subset of the compounds in the oncology and clinical drug collections, factors were identified that might lead to false-positive and false-negative scoring in the assay (Figure 3D and 3E).

One source of potential false positives for foci suppression at 2 hours was observed for compounds such as topotecan (Figure 3D), which was scored as 0.28. Visual inspection of the stored images revealed many small IRIF, though well below the minimal size threshold. We concluded that setting a lower threshold would still allow such compounds to be scored as hits, but the potential to mistake many small foci for failure to form IRIF argued for visual examination of each hit as a secondary screen. In turn, at the 24-hour time point, a wide range of GFP-IBD foci sizes and morphologies were observed that might skew counting (Figure 3E). Persistent foci might appear unchanged from their initial size and numbers (eg, deoxyadenosine), to form significantly larger foci (eg, synephrine), or to display large cell-to-cell variation in foci size and number (eg, cytarabine). Another confounding factor affecting foci numbers at 24 hours is that the kinetics and mechanisms of repair may depend on the phase of the cell cycle at the time of irradiation^34^ or other factors that lead to different rates of foci resolution between cells. Indeed, at 24 hours after IR in the controls, many cells have resolved their IRIF, but some retain multiple persistent IRIF. Compounds that primarily affect cell-cycle kinetics might lead to hits independent of their effects on DNA damage response. Again, visual examination of each hit was able to provide useful corroboration. We also considered that toxicity and/or cell death might alter apparent IRIF kinetics. However, because dead cells lose adhesion to the well surface, we found that following nuclei number/field as an indicator of cell health allowed reliable exclusion of the most toxic drugs when a cutoff of 0.5-fold change in nuclei number/field was applied.

To be inclusive of agents affecting both foci number and size and the limitations of detection and quantitation of GFP foci and definition of each Hoechst-stained nucleus, we used a fold change threshold of 0.5 at 2 hours to identify foci suppression. To accommodate the effects of agents that might have a cell-cycle phase-specific effect or other indirect effects, we adopted a criterion of 1.5-fold increase over control at 24 hours for foci persistence. These thresholds appeared sufficient to capture most compounds that change the kinetics or cause an alteration in pattern of foci, even in the presence of significant cell-to-cell variability.

Using our standard conditions and these thresholds, we assayed positive and negative controls to determine Z′ factor as a measure of reliability as a screening assay (see Materials and methods).^32^ The Z′ factor is a dimensionless statistic that reflects both the assay signal dynamic range and the variation associated with measurement. Typically, the Z′ factor is determined by distributing positive and negative controls over a screening plate and determining the reliability of the assay, which should give a very similar “low” value for the positive controls and a very similar “high” value for the negative controls. Here, a Z′ value of 0.5 would be considered adequate for screening, and a score of 1.0 an ideal assay. Thus, as an initial evaluation, we treated 48 wells of a 96-well plate with DMSO alone and 48 wells with DMSO plus 50 μM CGK733 for the 2-hour time point, or 48 wells with DMSO plus 25 μM etoposide for the 24-hour time point, incubated for 1 hour, irradiated with 6 Gy, and measured foci suppression at 2 hours or 24 hours using the granularity application module in MetaXpress. Subjecting these data to Z′ factor analysis yielded a value of >0.8 for both conditions, using a threshold of 0.5 at 2 hours to detect foci suppression and of 1.5 at 24 hours to detect foci persistence, establishing each as HTS-compatible assays.

### Screening collections of oncology and other drugs for IRIF formation and persistence

To further validate our approach as a tool for high-content screening of compound libraries, we performed two small model screens. We first examined 89 drugs of the DTP Approved Oncology Drugs Set. This library is heavily weighted toward DNA-damaging chemotherapy agents, including alkylators, demethylators, intercalators, DNA crosslinkers, and topoisomerase inhibitors. Based on extensive literature on the crosstalk between chemotherapy agents and radiation, many of these genotoxic agents would be expected to interfere with DNA repair, potentially resulting in increased foci persistence.

MCF7^Tet-On^ GFP-IBD cells were plated as described in Materials and methods and, after 48 hours, compounds were added at final concentrations of 6.25 μM and 50 μM in DMSO for 1 hour, and then cells were exposed to a single dose of 6 Gy. As internal controls for screening, we also added a column of eight control wells of negative (DMSO only) and positive controls (DMSO + 50 μM CGK733 or 25 μM etoposide) to each plate. When examined after 24 hours, 30 of the 89 drugs in this collection, or 33.7% of the total, qualified as hits in the assay at one or both concentrations (Table 1). Consistent with the high concentrations used for the screening, many of the other agents in the Approved Oncology Drugs Set led to significant decreases in cell number by 24 hours, as measured by Hoechst nuclei per field, and were excluded as toxic.

Among agents scored as hits from the Approved Oncology Drugs Set, many have already been examined as radiosensitizers (Table 2). Several of the alkylators, DNA repair inhibitors, the crosslinker bleomycin, three of the platinum compounds, and three topoisomerase type II inhibitors (mitoxandrone, teniposide, and etoposide) scored as hits. Several nucleoside analogues also scored highly, perhaps reflecting their direct or indirect effects on repair polymerases. However, other hits were less anticipated, representing potential false positives. Arsenic trioxide and a benzophenanthridine scoring as hits might reflect “off-target” effects at the concentrations used for screening. Interestingly, the spindle poison docetaxel got a high score, perhaps as a result of cell-cycle arrest rather than of any direct effect on DNA repair or chromatin modification. Nonetheless, taxanes are commonly combined with radiation and chemotherapy, often demonstrating significant synergy. On the other hand, failing to identify several genotoxic compounds from the Approved Oncology Drugs Set might be considered false negatives, based on their known activity as radiosensitizers in preclinical models or in cancer therapy. However, many of these agents were excluded as toxic, based on a decreased number of nuclei detected in treated wells. Capturing these agents might require screening at lower concentrations.

The NIH Clinical Collection contains 480 drugs and other agents directed at diverse targets with a history of use in human clinical trials. In this collection, 31 agents are classified as antineoplastic. The other major classes are central nervous system directed (136 agents), anti-infective (78), and cardiovascular (47). Screening the MCF7^Tet-On^ GFP-IBD cell line against this collection yielded 50 hits, or 10.4% of the total. Indeed, there was significant overlap of hits with the Approved Oncology Drugs Set, validating our screening approach. However, compounds from a wide range of drug classes also scored as hits, including many compounds with no known crosstalk with DNA metabolism or repair pathways or with known nuclear targets. Table 1 shows a distribution of targets or bioactivity of compounds that were positive in the screen.

Finally, to test our approach on a larger library, we screened the MCF7^Tet-On^ GFP-IBD cell line against the 2000-compound MicroSource Spectrum Collection. Drugs make up 50% of the collection with 800 compounds with US Adopted Name/US Pharmacopeia designations and 200 drugs limited to use in Europe. Thirty percent are natural products with unknown biological properties, and the remaining 20% are other bioactive agents representing nondrug enzyme inhibitors, receptor blockers, membrane active compounds, and toxins. Of the total, 121, or 6.3% of the compounds listed in this collection, are classified as antineoplastic by MicroSource. Each compound was added to a single well at 6.25 μM for 1 hour prior to irradiation, and then IRIF formation and persistence were determined 24 hours after 6 Gy irradiation. Eighty-four compounds made the cutoffs of >1.5 or <0.5 out of a total of 1922 wells screened, giving a 4.4% hit rate. Here, we obtained 67 hits that scored ≥1.5, indicating enhanced IRIF persistence, and 17 that scored ≤0.5, identifying compounds that either blocked IRIF formation, induced rapid resolution, or caused persistence of small IRIF below the threshold for detection. Again, a wide range of drug classes were identified, including many agents without previously described effects on DNA damage response.

**Figure 4:**
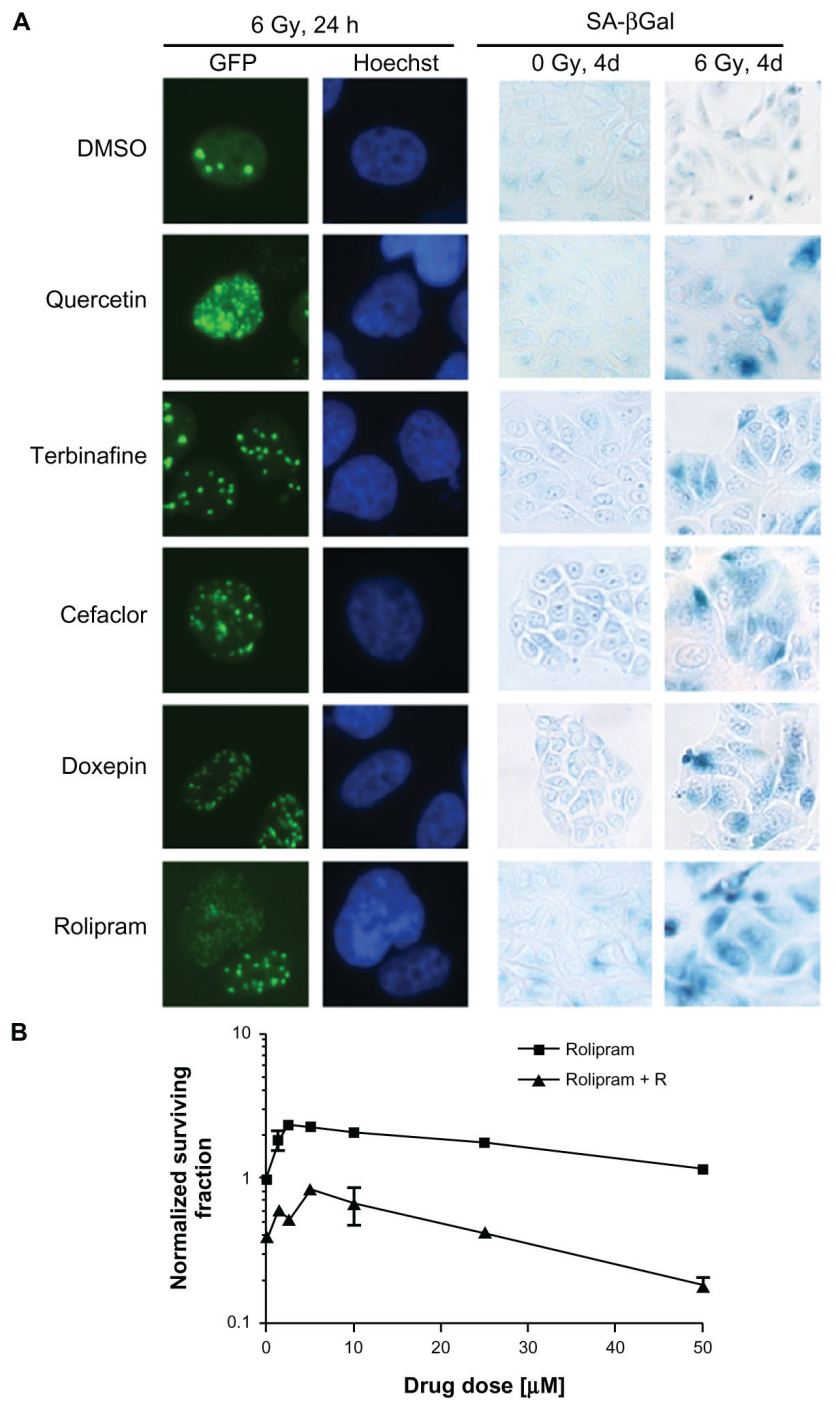
Secondary screening of agents that are not considered genotoxic that scored as hits from the foci persistence screen. **A**) Ionizing radiation-induced foci (IRIF) persistence and accelerated senescence activity of five selected hits that scored>1.5 at 6.25 μM or 50 μM. To evaluate IRIF persistence and senescence, McF7^Tet-On^ green fluorescent protein ionizing radiation-induced foci-binding domain (GFP-IBD) cells were plated in glass-bottom Petri dishes, treated with a range of concentrations of each agent for 1 hour, and then treated with 0 Gy or 6 Gy. Persistence of IRIF at 24 hours after 6 Gy IR is shown in the first and second columns, imaging GFP-IBD localization (GFP) and nuclear DNA (Hoechst) by fluorescence microscopy. Here, cells were pretreated with 50 μM quercetin, 1.25 μM terbinafine, 1.25 μM cefaclor, 1.25 μM doxepin, or 10 μM rolipram for 1 hour and imaged after 24 hours. Senescence enhancement is shown in the third and fourth columns by senescence-associated β-galactosidase (SA-βGal) staining and light microscopy. Here, cells were pretreated with 10 μM quercetin, 25 μM terbinafine, 25 μM cefaclor, 25 μM doxepin, or 10 μM rolipram for 1 hour, treated with 0 gy or 6 gy, and examined after 4 days. **B**) Clonogenic survival of MCF7^Tet-On^ gFP-IBD cells pretreated with increasing doses of rolipram 1 hour before treatment with 0 Gy or 2 Gy. Values are normalized to the efficiency of colony formation by untreated cells.

As a secondary screen, a selection of agents drawn from multiple drug classes and not considered DNA-damaging agents that had scored ≥1.5 in the GFP-IBD foci screen at 24 hours were obtained from commercial sources and assayed at a range of concentrations for enhanced foci persistence and increased senescence using standard methods. Five of six agents tested (the bioflavenoid quercetin, which had scored 1.52 at 6.25 μM; the allylamine antifungal terbinafine, 1.83 at 50 μM; the cephalosporin antibiotic cefaclor, 1.82 at 50 μM; the tricyclic antidepressant doxepin, 1.71 at 6.25 μM; and the antidepressant and anti-inflammatory rolipram, 1.62 at 50 μM) displayed low toxicity, increased persistent GFP foci, and enhanced SA-βGal staining compared with IR only (Figure 4A). These data suggest that automated analysis of GFP foci persistence can serve as a reliable screen for altered IRIF kinetics and, importantly, as a strong predictor of the potential to enhance the accelerated senescence response after irradiation.

Among the unexpected hits from the Clinical Collection and Spectrum Collection were several central nervous system-targeted drugs. As an example, we further examined rolipram, which has been described as a cyclic AMP phosphodiesterase-4 (PDE4) inhibitor. Rolipram can induce expression of G1 CDK inhibitors p21^Cip1^ and p27^Kip1^ to inhibit growth, induce differentiation, and cause apoptosis.^35^ In turn, rolipram increased survival of mice bearing intracranial U87 glioma xenografts after treatment with temozolomide and IR, though not with IR only.^36^ In our study, pretreatment with rolipram enhanced accelerated senescence after irradiation suggesting that rolipram might also confer radiosensitization. Thus, we assayed clonogenic survival after MCF7^Tet-On^ GFP-IBD cells were treated for 1 hour with 0–50 μM rolipram and then irradiated at the clinically relevant dose of 2 Gy. Colony formation after pretreatment with rolipram and then a single dose of 0 Gy or 2 Gy demonstrated a complex dose-response pattern (Figure 4B), but radiosensitization greater than additive was observed at 25 μM and 50 μM rolipram.

## Discussion

We report a novel high-throughput, high-content screening method directed at discovering novel radiosensitizers via live cell imaging. Our approach relies on irradiating cells expressing a GFP fusion to the IRIF IBD of the checkpoint and repair protein 53BP1 and following relocalization of fluorescent signal as a reporter for IRIF. As we recently showed,^31^ this fusion protein, GFP-IBD, reliably relocalizes to sites of DNA damage after irradiation. In our prior work, we examined the effects of treating cells expressing GFP-IBD with a poly(ADP-ribose) polymerase inhibitor, veliparib (ABT-888).^37,38^ This DNA repair inhibitor caused marked GFP-IBD foci persistence, significant radiosensitization, and accelerated cell senescence. Our prior studies combined with recent work by others^16,18,20,22,27,29,39^ suggest the hypothesis that identifying agents that can promote IRIF persistence might offer a general route to radiosensitization via enhancing accelerated (therapy-induced) senescence. Here, we have extended this work to develop a high-content screening assay that has revealed additional compounds that may target one or more steps in the detection and repair of DNA DSBs and thereby alter the function of IRIF.

Our prior work^31^ and the results reported here confirm that DNA damage and repair have a direct effect on foci number but suggest that a range of other factors may also determine the kinetics of foci persistence and growth. We previously found that after forming rapidly, IRIF decrease significantly in number during the first hour after irradiation before entering a longer, slower phase of foci resolution.^31^ This initial phase presumably reflects rapid, nonhomologous end joining, whereas the slower phase may reflect delayed rejoining activity as well as homology-dependent DSB repair mechanisms such as homologous recombination. However, the relationship between DNA repair and IRIF formation, size, and resolution is not direct. Indeed, the growth in size of IRIF over time appears to be highly sensitive to multiple influences and may, in part, involve fusion of initial foci.^10,40^ As such, our choice to examine foci at 2 hours and 24 hours afforded us the potential to detect small molecules targeting multiple different processes affecting DNA repair and chromatin function.

Chemical radiosensitization has been the subject of multiple recent reviews.^41–46^ Translated to the clinic,^47^ many patients are now treated with IR combined with chemotherapy agents that induce distinct forms of DNA damage, such as DNA polymerase inhibitors, alkylators, or topoisomerase inhibitors. However, normal tissue toxicity is often dose limiting. The rapid growth of knowledge regarding the pathways mediating DNA damage responses and cell death^48^ has led to efforts at mechanism-based drug discovery to identify agents that are toxic only to cells within the radiation field. Radiosensitizers that target DNA repair^8,49–51^ and DNA damage checkpoint signaling,^52,53^ affecting cell death or tumor cell senescence,^18–20^ have been described. Ewald et al^54^ have described a successful high-throughput screen for small-molecule inducers of accelerated senescence that identified four new compounds from three small-molecule libraries totaling 4160 compounds. Both biologically targeted and untargeted agents have shown promise in IR sensitization of cancer cells in preclinical models, but agents lacking cytotoxic activity on their own have yet to demonstrate clinical impact.

Based on these considerations, we adapted analysis of IRIF formation and resolution to a 96-well screening format. By optimizing factors that contributed to well-to-well variability, including cell culture conditions, induction of the GFP reporter, IR dose, and assay timing, we were able to obtain sufficient reproducibility to achieve Z ′ numbers >0.8 both for suppression of foci at 2 hours and persistence of foci at 24 hours, using the ATM inhibitor CGK733 and the type II topoisomerase inhibitor etoposide as the respective positive controls. Reflecting our goal of identifying radiosensitizers that target IRIF dynamics, we chose to expose cells to compounds for only 1 hour prior to irradiation and did not remove the compounds from wells during the subsequent 24-hour screening period. We identified 144 drugs that promote IRIF persistence from a total of 2459 screened from three collections. This might seem an unexpectedly high number of hits obtained from a small number of compounds. We have identified several factors that contributed to this result. One is that each of the three libraries contained a significant number of genotoxic drugs already known to enhance the effects of IR, and these provided a list of expected hits. Indeed, several of the agents are used in current chemoradiation therapy protocols.^47^ Although a significant fraction of the 144 hits are these genotoxic drugs, other agents, particularly among the hits from the NIH Clinical Collection, were completely unanticipated, considering their applications have previously been restricted to treatment of diseases unrelated to cancer. Most have not previously been identified as affecting DNA repair and are not considered genotoxic on their own. Here, we ascribe the hits to factors such as the relatively high concentration of agents used in screening and the potentially very large number of molecular targets that comprise the IRIF itself and the pathways of DNA repair and chromatin modification that must be active to allow resolution of foci after irradiation.

Although the goal of many small-molecule screens is discovery of new molecular entities, the high costs for hit to lead, preclinical and clinical development, along with the remarkably low success rate of reaching the market make this route unattractive except to large pharmaceutical firms with resources to maintain a pipeline. As an alternative, the concept of repurposing or repositioning continues to gain traction.^55,56^ Typically, repurposing screens are limited to collections of compounds already tested in animals or humans, including investigational, current, and expired small-molecule drugs and natural products. As such, hits are likely to satisfy criteria for low toxicity and drug-like chemistry^57^ and can provide future leads for repurposing as nontoxic radiosensitizers. Where the compound displays its desirable off-target effects only at a high dose, selective optimization of side activities^58^ may provide a cost-effective route to optimization, perhaps only requiring screening of existing structure–activity relationship libraries.

At least some of the compounds that induced IRIF persistence also accelerated senescence of the MCF7^Tet-On^ GFP-IBD cells. Screening compound libraries for IRIF persistence in living cells is likely to offer a profitable approach for the identification of additional nontoxic compounds that enhance accelerated senescence after genotoxic therapy of cancer cells. These or other agents that can promote IRIF persistence might offer a general route to radiosensitization via enhanced senescence. Our future efforts will be directed at identifying the most promising hits for further development and translating the testing to animal models and clinical trials.

## Figures and Tables

**Table 1:** Classification of hits by target or bioactivity in the screen of two libraries

Target or bioactivity	Number of compounds	
	Clinical collection	Approved oncology drugs set
DNA damage/repair	7	21
Hormones	6	2
Psychoactive drugs	14	0
Tyrosine kinase inhibitors	0	4
Ion channel inhibitors	3	0
Antihistamines	4	0
Target adenosine receptor	3	0
Target adrenergic receptor	2	0
Unclassified	11	3

**Table 2:** Ionizing radiation-induced foci (IRIF)/cell relative to 6 Gy IR plus dimethyl sulfoxide (DMSO) control at 24-hour time point for drugs classified as antineoplastics in the approved oncology drugs set screen at two concentrations

DNA-damaging agent	Mechanism of action	IRIF/cell relative to IR + DMSO	
		50 μM	6.25 μM
Temozolomide	Base alkylation	2.57	1.04
Pipobroman	Base alkylation	2.55	1.25
Thiotepa	Base alkylation	2.43	1.50
Chlorambucil	Base alkylation	2.30	1.30
Benzophenanthridine	Topoisomerase II inhibitor	2.36	0.60
Teniposide	Topoisomerase II inhibitor	1.66	0.93
Etoposide	Topoisomerase II inhibitor	0.41	1.95
Mitoxantrone	Topoisomerase II inhibitor	Toxic	0.20
Cisplatin	Intrastrand crosslinker	2.12	0.93
Oxaliplatin	Intrastrand crosslinker	1.93	1.08
Carboplatin	Intrastrand crosslinker	1.59	1.26
Bleomycin	Interstrand crosslinker	3.31	1.71
Thioguanine	Purine synthesis inhibitor	3.39	1.60
Pentostatin	Adenosine deaminase inhibitor	1.57	0.63
Cladribine	Adenosine deaminase inhibitor	1.65	1.42
Deoxyadenosine	Adenosine deaminase inhibitor	1.75	1.17
Doxorubicin	Alkylator	1.12	2.12
Valrubicin	Alkylator	1.88	1.46
Cytaribine	DNA polymerase inhibitor	2.42	1.92
Docetaxel	Tubulin stabilizer	1.66	1.38
Arsenic trioxide	Thioredoxin reductase inhibitor	3.42	1.54

## References

[1] Van Attikum H, Gasser SM (2009). Crosstalk between histone modifications during the DNA damage response. Trends Cell Biol.

[2] Corpet A, Almouzni G (2009). A histone code for the DNA damage response in mammalian cells?. The EMBO Journal.

[3] Rossetto D, Truman AW, Kron SJ, Cote J (2010). Epigenetic modifications in double-strand break DNA damage signaling and repair. Clin Cancer Res.

[4] Hollick JJ, Rigoreau LJ, Cano-Soumillac C (2007). Pyranone, thiopyranone, and pyridone inhibitors of phosphatidylinositol 3-kinase related kinases. Structure-activity relationships for DNA-dependent protein kinase inhibition, and identification of the first potent and selective inhibitor of the ataxia telangiectasia mutated kinase. J Med Chem.

[5] Peterson S, Wang L, Robertson K (2009). Reply to “Corrected structure of mirin, a small-molecule inhibitor of the Mre11–Rad50–Nbs1 complex”. Nat Chem Biol.

[6] Garner K, Pletnev A, Eastman A (2009). Corrected structure of mirin, a small-molecule inhibitor of the Mre11–Rad50–Nbs1 complex. Nat Chem Biol.

[7] Dupré A, Boyer-Chatenet L, Sattler R (2008). A forward chemical genetic screen reveals an inhibitor of the Mre11–Rad50–Nbs1 complex. Nat Chem Biol.

[8] Won J, Kim M, Kim N (2006). Small molecule-based reversible reprogramming of cellular lifespan. Nat Chem Biol.

[9] Bonner WM, Redon CE, Dickey JS (2008). GammaH2AX and cancer. Nat Rev Cancer.

[10] Belyaev IY (2010). Radiation-induced DNA repair foci: spatio-temporal aspects of formation, application for assessment of radiosensitivity and biological dosimetry. Mutat Res.

[11] Yamauchi M, Oka Y, Yamamoto M (2008). Growth of persistent foci of DNA damage checkpoint factors is essential for amplification of G1 checkpoint signaling. DNA Repair (Amst).

[12] Liu SK, Olive PL, Bristow RG (2008). Biomarkers for DNA DSB inhibitors and radiotherapy clinical trials. Cancer Metastasis Rev.

[13] Banath JP, Klokov D, MacPhail SH (2010). Residual gammaH2AX foci as an indication of lethal DNA lesions. BMC Cancer.

[14] Camphausen K, Burgan W, Cerra M (2004). Enhanced radiation-induced cell killing and prolongation of gammaH2AX foci expression by the histone deacetylase inhibitor MS-275. Cancer Res.

[15] Taneja N, Davis M, Choy JS (2004). Histone H2AX phosphorylation as a predictor of radiosensitivity and target for radiotherapy. J Biol Chem.

[16] Rodier F, Coppe JP, Patil CK (2009). Persistent DNA damage signalling triggers senescence-associated inflammatory cytokine secretion. Nat Cell Biol.

[17] Nakamura AJ, Chiang YJ, Hathcock KS (2008). Both telomeric and non-telomeric DNA damage are determinants of mammalian cellular senescence. Epigenetics Chromatin.

[18] D’Adda di Fagagna F (2008). Living on a break: cellular senescence as a DNA-damage response. Nat Rev Cancer.

[19] Roninson IB, Broude EV, Chang BD (2001). If not apoptosis, then what? Treatment-induced senescence and mitotic catastrophe in tumor cells. Drug Resist Updat.

[20] Ewald JA, Desotelle JA, Wilding G, Jarrard DF (2010). Therapy-induced senescence in cancer. J Natl Cancer Inst.

[21] Schmitt C (2007). Cellular senescence and cancer treatment. BBA-Reviews on Cancer.

[22] Bilsland AE, Keith WN, Adams PD, Sedivy JM (2010). Mining cellular senescence for drug targets. Cellular Senescence and Tumor Suppression.

[23] Roninson IB (2003). Tumor cell senescence in cancer treatment. Cancer Res.

[24] Xue W, Zender L, Miething C (2007). Senescence and tumour clearance is triggered by p53 restoration in murine liver carcinomas. Nature.

[25] Krizhanovsky V, Yon M, Dickins RA (2008). Senescence of activated stellate cells limits liver fibrosis. Cell.

[26] Kuilman T, Michaloglou C, Mooi WJ, Peeper DS (2010). The essence of senescence. Genes Dev.

[27] Gewirtz DA, Holt SE, Elmore LW (2008). Accelerated senescence: an emerging role in tumor cell response to chemotherapy and radiation. Biochem Pharmacol.

[28] Kim K, Pollard J, Norris A (2009). High-throughput screening identifies two classes of antibiotics as radioprotectors: tetracyclines and fluoroquinolones. Clin Cancer Res.

[29] Lawless C, Wang C, Jurk D (2010). Quantitative assessment of markers for cell senescence. Exp Gerontol.

[30] Huyen Y, Zgheib O, Ditullio RA (2004). Methylated lysine 79 of histone H3 targets 53BP1 to DNA double-strand breaks. Nature.

[31] Efimova EV, Mauceri HJ, Golden DW (2010). Poly(ADP-ribose) polymerase inhibitor induces accelerated senescence in irradiated breast cancer cells and tumors. Cancer Res.

[32] Zhang JH, Chung TD, Oldenburg KR (1999). A simple statistical parameter for use in evaluation and validation of high throughput screening assays. J Biomol Screen.

[33] Yoo E, Kim BU, Lee SY (2005). 53BP1 is associated with replication protein A and is required for RPA2 hyperphosphorylation following DNA damage. Oncogene.

[34] Branzei D, Foiani M (2008). Regulation of DNA repair throughout the cell cycle. Nat Rev Mol Cell Biol.

[35] Chen TC, Wadsten P, Su S (2002). The type IV phosphodiesterase inhibitor rolipram induces expression of the cell cycle inhibitors p21(Cip1) and p27(Kip1), resulting in growth inhibition, increased differentiation, and subsequent apoptosis of malignant A-172 glioma cells. Cancer Biol Ther.

[36] Goldhoff P, Warrington NM, Limbrick DD (2008). Targeted inhibition of cyclic AMP phosphodiesterase-4 promotes brain tumor regression. Clin Cancer Res.

[37] Penning TD, Zhu GD, Gandhi VB (2009). Discovery of the poly(ADP-ribose) polymerase (PARP) inhibitor 2-[(R)-2-methylpyrrolidin-2-yl]-1H-benzimidazole-4-carboxamide (ABT-888) for the treatment of cancer. J Med Chem.

[38] Donawho CK, Luo Y, Penning TD (2007). ABT-888, an orally active poly(ADP-ribose) polymerase inhibitor that potentiates DNA-damaging agents in preclinical tumor models. Clin Cancer Res.

[39] Campisi J, d’Adda di Fagagna F (2007). Cellular senescence: when bad things happen to good cells. Nat Rev Mol Cell Biol.

[40] Falk M, Lukasova E, Kozubek S (2010). Higher-order chromatin structure in DSB induction, repair and misrepair. Mutat Res.

[41] Katz D, Ito E, Liu FF (2009). On the path to seeking novel radiosensitizers. Int J Radiat Oncol Biol Phys.

[42] Wardman P (2007). Chemical radiosensitizers for use in radiotherapy. Clin Oncol (R Coll Radiol).

[43] Michod D, Widmann C (2007). DNA-damage sensitizers: potential new therapeutical tools to improve chemotherapy. Critical Reviews in Oncology and Hematology.

[44] Bolderson E, Richard D, Zhou B, Khanna K (2009). Recent advances in cancer therapy targeting proteins involved in DNA double-strand break repair. Clin Cancer Res.

[45] Tofilon PJ, Camphausen K (2009). Molecular targets for tumor radiosensitization. Chem Rev.

[46] Zhu Y, Hu J, Hu Y, Liu W (2009). Targeting DNA repair pathways: A novel approach to reduce cancer therapeutic resistance. Cancer Treat Rev.

[47] Seiwert TY, Salama JK, Vokes EE (2007). The concurrent chemoradiation paradigm: general principles. Nat Clin Pract Oncol.

[48] Jackson S, Bartek J (2009). The DNA-damage response in human biology and disease. Nature.

[49] Damia G, D’Incalci M (2007). Targeting DNA repair as a promising approach in cancer therapy. Eur J Cancer.

[50] Helleday T, Petermann E, Lundin C (2008). DNA repair pathways as targets for cancer therapy. Nat Rev Cancer.

[51] O’Connor MJ, Martin NM, Smith GC (2007). Targeted cancer therapies based on the inhibition of DNA strand break repair. Oncogene.

[52] Zhou BB, Sausville EA (2003). Drug discovery targeting Chk1 and Chk2 kinases. Prog Cell Cycle Res.

[53] Luo Y, Leverson JD (2005). New opportunities in chemosensitization and radiosensitization: modulating the DNA-damage response. Expert Rev Anticancer Ther.

[54] Ewald JA, Peters N, Desotelle JA (2009). A high-throughput method to identify novel senescence-inducing compounds. J Biomol Screen.

[55] Chong C, Sullivan D (2007). New uses for old drugs. Nature.

[56] Ashburn T, Thor K (2004). Drug repositioning: identifying and developing new uses for existing drugs. Nat Rev Drug Discov.

[57] Lipinski C, Lombardo F, Dominy B, Feeney P (1997). Experimental and computational approaches to estimate solubility and permeability in drug discovery and development settings. Adv Drug Deliv Rev.

[58] Wermuth C (2006). Selective optimization of side activities: the SOSA approach. Drug Discov Today.

